# Positive Association of Serum Galectin-3 with the Development of Aortic Stiffness of Patients on Peritoneal Dialysis

**DOI:** 10.3390/jcm12103519

**Published:** 2023-05-17

**Authors:** Po-Yu Huang, Chen-Sen Huang, Yu-Li Lin, Yi-Hsin Chen, Szu-Chun Hung, Jen-Pi Tsai, Bang-Gee Hsu

**Affiliations:** 1 Division of Nephrology, Department of Internal Medicine, Dalin Tzu Chi Hospital, Buddhist Tzu Chi Medical Foundation, Chiayi 62247, Taiwan; poyuhs13628@gmail.com (P.-Y.H.); dm839796@tzuchi.com.tw (C.-S.H.); 2 School of Medicine, Tzu Chi University, Hualien 97004, Taiwan; nomo8931126@gmail.com (Y.-L.L.); nephp06@gmail.com (Y.-H.C.); szuchun.hung@gmail.com (S.-C.H.); 3 Division of Nephrology, Hualien Tzu Chi Hospital, Buddhist Tzu Chi Medical Foundation, Hualien 97004, Taiwan; 4 Division of Nephrology, Department of Internal Medicine, Taichung Tzu Chi Hospital, Buddhist Tzu Chi Medical Foundation, Taichung 40201, Taiwan; 5 Division of Nephrology, Department of Internal Medicine, Taipei Tzu Chi Hospital, Buddhist Tzu Chi Medical Foundation, Taipei 23142, Taiwan

**Keywords:** carotid–femoral pulse wave velocity, aortic stiffness, peritoneal dialysis, galectin-3

## Abstract

A novel cardiovascular stress biomarker known as galectin-3 might be useful for anticipating adverse cardiovascular outcomes. The objective of the current investigation was to assess the association between serum galectin-3 levels and aortic stiffness (AS) in 196 patients on peritoneal dialysis. An enzyme-linked immunosorbent examination and a cuff-based volumetric displacement were employed to determine the levels of serum galectin-3 and the carotid-femoral pulse wave velocity (cfPWV), respectively. The AS group had 48 patients in total (24.5%) with cfPWV greater than 10 m/s. The AS group, when compared with the group without AS, had a significantly higher prevalence of diabetes mellitus and hypertension in addition to greater fasting glucose levels, waist circumference, systolic blood pressure, and serum galectin-3 levels. Multivariate logistic and linear regression analysis demonstrated that serum glactin-3 levels, in addition to gender and age, were significantly and independently associated with cfPWV and AS. Serum galectin-3 levels were linked with AS, according to a receiver operating characteristic curve analysis, with an area under the curve of 0.648 (95% confidence interval, 0.576–0.714; *p* = 0.0018). In summary, there was a significant correlation between serum galectin-3 levels and cfPWV in patients undergoing peritoneal dialysis therapy for end-stage kidney disease.

## 1. Introduction

In both the general population and people with end-stage kidney disease (ESKD), aortic stiffness (AS) is a recognized risk factor and biomarker for cardiovascular (CV) events, CV mortality, and all-cause mortality [1,2,3]. In patients receiving maintenance peritoneal dialysis (PD), AS independently predicts fatal and nonfatal unfavorable CV outcomes, according to several cohort studies [4,5]. The gold-standard strategy for evaluating AS at the present time is carotid-femoral pulse wave velocity (cfPWV), which has been demonstrated to be useful in predicting adverse CV events and mortality [6,7].

Several organs and tissues, including the lung, liver, gastrointestinal tract, kidney, and heart, express galectin-3, a beta-galactoside-binding lectin with a molecular weight of about 30 kDa. Galactin-3 is mainly found in the cytoplasm, where it participates in intracellular signaling. It is also found in the nucleus, on the cell surface, and in the extracellular matrix. Inflammation and injury to tissues cause galectin-3 to be expressed, which eventually leads to organ fibrosis and malfunction [8,9]. Galectin-3 is a potential biomarker of clinical outcomes in acute and chronic heart failure [10]. Furthermore, an association has been reported between serum galectin-3 levels and kidney disease. Galectin-3 contributes to the normal process of nephrogenesis but also has a role in kidney fibrosis and kidney dysfunction [11]. Urinary galactin-3 levels reportedly have utility in identifying patients at increased risk of kidney disease progression [12]. Serum galectin-3 concentrations increase as the glomerular filtration rate declines. In patients with chronic kidney disease (CKD) and on dialysis, galectin-3 could predict cardiovascular morbidity, infection-related death, and all-cause mortality [13].

The association between serum galectin-3 levels and AS has previously been studied. In one cross-sectional study focusing on older patients with a mean age of 75 years, greater serum galectin-3 levels were correlated with greater central AS measured by cfPWV. However, the association was attenuated after adjustment for certain CV risk factors and biomarkers, including diabetes mellitus (DM), hypertension (HTN), dyslipidemia, estimated glomerular filtration rate, smoking, and serum high-sensitivity C-reactive protein (CRP) levels [14]. In another cross-sectional investigation, patients with ESKD receiving maintenance hemodialysis had their serum galectin-3 levels compared to AS. Even after adjusting for age, serum albumin, serum phosphorus, DM, blood pressures, serum low-density lipoprotein, serum CRP, and dialysis efficiency in this investigation, galectin-3 was still found to be independently associated with AS [15]. A positive association between galectin-3 and cfPWV was also concluded in a pilot study, which focused on patients with chronic ischemic heart disease and a reduced ventricular ejection fraction [16].

Because CV events are the leading cause of death in patients receiving PD and the role of galectin-3 in the development of AS in these patients has not yet been thoroughly clarified, the current investigation attempted to explore the relationship between serum galectin-3 levels and AS in this patient population.

## 2. Materials and Methods

### 2.1. Participants

We performed a cross-sectional study in four Tzu Chi hospitals in Taiwan: Hualien, Taipei, Taichung, and Dalin Tzu Chi Hospital. This study received approval from the Hualien Tzu Chi Hospital, Buddhist Tzu Chi Medical Foundation’s research ethics committee (IRB108-219-A). A total of 196 individuals with ESKD who had been using maintenance PD for at least three months were enrolled in the trial between February 2020 and May 2021. In the study, 124 patients received automated PD (APD), whereas the remaining 72 patients received continuous ambulatory PD (CAPD). Underlying active infections, cancer, severe heart failure, acute coronary syndrome, stroke, previous limb amputations, and an incapacity to give informed consent were considered as research exclusion criteria.

Medical records were used to gather information on solute clearance and the adequacy of dialysis, including total and peritoneal creatinine clearance as well as the weekly and peritoneal fractional clearance index for urea (Kt/V). After a 10 min rest period, trained personnel used standard mercury sphygmomanometers with the proper cuff size to take triplicate readings of the systolic (SBP) and diastolic (DBP) blood pressures. A SBP of 140 mm Hg or more, a DBP of 90 mm Hg or more, and/or the use of antihypertensive medications during the previous two weeks were all considered to be indicators of HTN. A fasting plasma glucose level of greater than 126 mg/dL and/or regular use of oral diabetic medications and/or insulin were used for the identification of DM.

### 2.2. Anthropometric Analyses

Patients were dressed in light clothing when body weights were measured. Patients were asked to stand barefoot or in stockings while heights were measured. Weight/height^2^ (kg/m^2^) was used to compute the body mass index (BMI) [17].

### 2.3. Analyses of the Biochemistry

Each participant had a 5 mL overnight (more than 8 h) fasting blood sample taken before the next morning’s dialysate exchange. Immediately after collection, samples were centrifuged at 3000× *g* for 10 min. Within an hour of collection, serum was transported for biochemical evaluation and stored at 4 °C. A Siemens Advia 1800 autoanalyzer from Siemens Healthcare GmbH in Henkestr, Germany, was used to analyze the serum for blood urea nitrogen, creatinine, total calcium, phosphorus, albumin, fasting glucose, and total cholesterol [17]. By using commercially available enzyme-linked immunosorbent assays, the serum concentrations of galectin-3 (RayBiotech, Peachtree Corners, GA, USA) and intact parathyroid hormone (iPTH, IBL International GmbH, Hamburg, Germany) were measured [18].

### 2.4. Measurement of Carotid–Femoral Pulse Wave Velocity

Measurements of cfPWV to assess AS were performed using cuff-based volumetric displacement (SphygmoCor XCEL, AtCor Medical, Sydney, NSW, Australia) [12]. Participants rested for a minimum of 10 min before measurements were taken in the morning while they were lying supine in a quiet, temperature-controlled environment. Briefly, the cuff of the XCEL device was placed on the left upper arm and brachial SBP and DBP were automatically recorded using standard oscillometric measurements followed by immediate reinflation of the cuff to a subdiastolic pressure level. The XCEL system substitutes volumetric displacement waveform from an upper thigh cuff for femoral artery tonometry to measure cfPWV, while tonometry is employed to measure the carotid pulse [19]. A cfPWV of greater than 10 m/s implies AS, according to an expert consensus guideline [20,21]. Accordingly, the group without AS in the present study comprised patients with a cfPWV ≤ 10 m/s.

### 2.5. Statistical Analyses

The Kolmogorov–Smirnov test was used to determine if continuous variables were normally distributed. The two-tailed independent Student’s *t*-test was used to compare normally distributed continuous variables between two groups. These variables were reported as mean ± standard deviation. The Mann–Whitney U test was used to compare non-normally distributed variables between groups. Non-normally distributed variables were expressed as median and interquartile ranges. Prior to the linear regression analysis, non-normally distributed data were logarithmically converted. The χ^2^ test was used to assess categorical data, which were expressed as numbers (percentages). Univariate logistic regression analysis was applied to assess the risk factors for the development of AS. Afterwards, we incorporated those factors that showed significance (female, diabetes, hypertension, waist circumference, age, systolic blood pressure, fasting glucose and galectin-3) into a multivariate logistic regression analysis to determine the correlates of AS. Univariable and multiple linear regression was used to also assess the correlates of galectin-3. The C-statistic was further adopted as a discriminatory test for analyzing the role of galectin-3 in correlation with AS. The area under the curve (AUC) was computed using a receiver operating curve to determine the optimal cutoff of the serum galectin-3 value, so as to discriminate patients with AS from those without AS. SPSS for Windows (version 19.0; SPSS Inc., Chicago, IL, USA) was used to analyze the data. Statistical significance was determined by *p* values less than 0.05.

## 3. Results

The clinical characteristics of the 196 patients receiving PD are listed in Table 1. Comorbidities included DM (*n* = 77; 39.3%) and HTN (*n* = 140; 71.4%). Forty-eight patients (24.5%) were assigned to the AS group. Compared to those not having AS, patients with AS had older age (*p* = 0.006), greater waist circumference (*p* = 0.002), higher SBP (*p* = 0.038), greater fasting glucose levels (*p* < 0.001), a higher prevalence of DM (*p* < 0.001) and HTN (*p* = 0.036), greater serum galectin-3 levels (*p* = 0.001), and were less likely to be female (*p* < 0.001). Serum total cholesterol, albumin, calcium, phosphorus, and iPTH levels, BMI, total and dialysate solute clearance, and proportions of patients receiving angiotensin-receptor blockers, beta blockers, and calcium-channel blockers were similar between the two groups.

Multivariate logistic regression analysis showed that higher serum galectin-3 levels (odds ratio (OR), 1.029; 95% confidence interval (CI), 1.002–1.057; *p* = 0.034)), female gender (OR, 0.287; 95% CI, 0.128–0.646; *p* = 0.003) and older age (OR, 1.044; 95% CI, 1.011–1.078; *p* = 0.008) as independent risk factors for the development of AS among study participants (Table 2).

Simple linear regression analysis demonstrated that cfPWV was negatively correlated with female gender (*r* = −0.330; *p* < 0.001) and positively correlated with DM (*r* = 0.387; *p* < 0.001), age (*r* = 0.338; *p* < 0.001), BMI (*r* = 0.216; *p* = 0.002), waist circumference (*r* = 0.332; *p* < 0.001), SBP (*r* = 0.250; *p* < 0.001), logarithmically transformed (log) serum glucose levels (*r* = 0.372; *p* < 0.001), and serum galectin-3 levels (*r* = 0.274; *p* < 0.001). After adjustment for covariates with multivariate forward stepwise linear regression analysis, female gender (β = –0.309; adjusted R^2^ change = 0.089; *p* < 0.001), DM (β = 0.257; adjusted R^2^ change = 0.161; *p* < 0.001), age (β = 0.306; adjusted R^2^ change = 0.082; *p* < 0.001), SBP (β = 0.184; adjusted R^2^ change = 0.028; *p* = 0.002), and serum galectin-3 levels (β = 0.184; adjusted R^2^ change = 0.031; *p* = 0.001) were found to be independently correlated with cfPWV (Table 3).

Another regression analysis revealed the correlation between serum galectin-3 concentrations and certain clinical variables. DM (*r* = 0.146; *p* = 0.041), HTN (*r* = 0.160; *p* = 0.025), cfPWV (*r* = 0.212; *p* = 0.003), log-glucose (*r* = 0.161; *p* = 0.024), and peritoneal Kt/V (*r* = 0.146; *p* = 0.041) were positively correlated with galectin-3, which was noted from the simple linear regression analysis. Multivariate forward stepwise linear regression analysis concluded that only PWV was independently associated with galectin-3 (Table 4).

**Figure 1:**
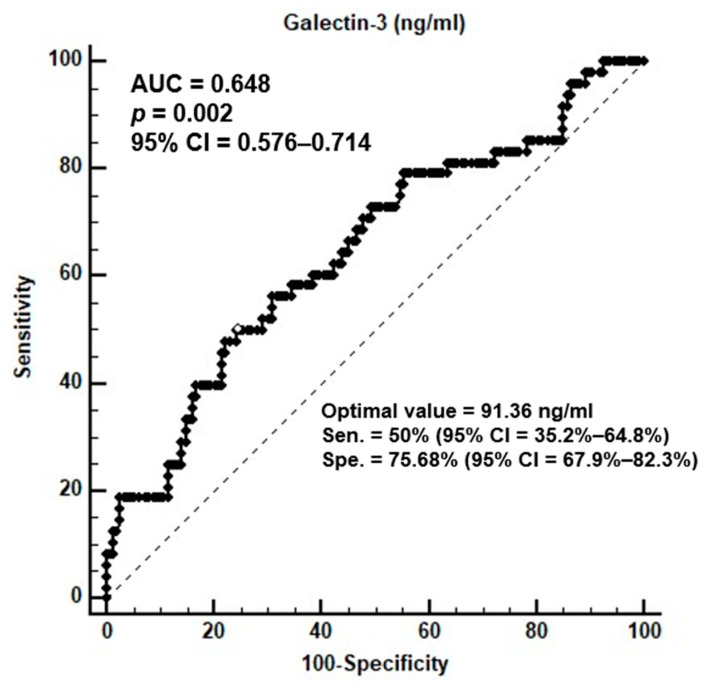
The diagnostic efficacy of galectin-3 levels for predicting AS among 196 patients on peritoneal dialysis.

The C-statistic discrimination tests listed in Supplementary Table S1 revealed that the C-statistic insignificantly increased (from 0.778 to 0.795; *p* = 0.318) after addition of galectin-3 to certain clinical variables, including age, gender, SBP, and DM. As shown in Figure 1, the optimal serum galectin-3 level to predict AS based on the area under the receiver operating characteristic curve (AUC, 0.648; 95% CI, 0.576–0.714; *p* = 0.0018) was 91.36 ng/mL, with a sensitivity of 50%, specificity of 75.7%, positive predictive value of 40.0%, and negative predictive value of 82.4%.

## 4. Discussion

The primary finding of this study was that serum galectin-3 levels were independently associated with AS; other independent correlates of AS were male gender, DM, age, and SBP. Serum galectin-3 appears to be useful as a distinctive biomarker for the prediction of AS in patients on maintenance PD, in addition to male gender and older age. On the other hand, although underlying HTN and hyperglycemia were associated with galectin-3, the association was attenuated after adjustment of certain variables.

The mechanisms underlying vascular stiffening involve activation of the renin–angiotensin system along with vascular smooth muscle cell (VSMC) proliferation, inflammation, oxidative stress, increased collagen content in the extracellular matrix, insulin resistance, oxidized low-density lipoprotein (LDL), mechanical signal transduction, and genetic and epigenetic factors [22,23]. In chronic kidney disease (CKD) populations, besides the aforementioned pathways, uremic toxins and dialysis-specific factors also contribute. There is disturbed balance between inducers (for example, serum calcium and phosphorus, advanced glycation end-products, parathyroid hormone, cytokines, and uremic toxins including indoxyl sulfate and p-cresyl sulfate) and inhibitors (fetuin-A, osteopontin, osteoprotegerin, matrix Gla protein, and bone morphogenetic protein 7) of blood vessel calcification. The vasoactive molecule endothelin-1 worsens vascular calcification by acting via ETA receptors. Furthermore, PD fluid contains high concentrations of glucose as well as glucose degradation products, which are directly linked to chronic inflammation, oxidative stress, and a predisposition to arterial stiffening. Dialysis catheters are foreign bodies and may enhance the inflammatory reactions, eventually triggering vascular remodeling [24].

Galectin-3 has a critical role in vascular remodeling and atherosclerosis through a variety of mechanisms. Galectin-3 upregulates the expression and secretion of proinflammatory cytokines, including interleukin-6, interleukin-1β, and tumor necrosis factor-α. Galectin-3 also induces the production of chemotactic factors such as C-C chemokine ligand 2, 3, 5, and 8, and C-X-C motif chemokine 8. As a result, galectin-3 is a critical regulator of inflammatory pathways [25,26]. Galectin-3 promotes the release of reactive oxygen species via activation of nicotinamide adenine dinucleotide phosphate (NADPH) oxidase; thus, galectin-3 is directly linked to oxidative stress [27]. Additionally, galectin-3 contributes to the processes of atherosclerosis and arterial stiffening by impairing normal endothelial function, causing foam cell formation, and leading to VSMC proliferation and migration [25,28]. As galectin-3 expression is reportedly increased in vulnerable atherosclerotic plaques [26], galectin-3 knockout mice or mice undergoing galectin-3 inhibition had smaller atherosclerotic plaque areas and reduced lipid cores [29]. In rat models of pulmonary hypertension, increased galectin-3 expression has been observed in VSMCs, with galectin-3 inhibitors shown to slow disease progression [30,31]. In summary, galectin-3 may contribute to atherosclerotic changes and the development of AS.

DM was significantly associated with AS in the present study. Our results also revealed a correlation between galectin-3 and underlying DM as well as fasting plasma glucose, although the association was attenuated after usage of multivariate regression analysis by adjusting other clinical parameters. PWV and pulse pressures are significantly increased in patients with DM, and have been reported to predict adverse CV outcomes. Increased PWV and pulse pressure are associated with markers of end-organ dysfunction, including albuminuria, retinopathy, and nephropathy [32]. Insulin resistance is associated with decreased nitric oxide-dependent vasodilation, increased neointimal hyperplasia, and increased VSMC proliferation and migration [33,34]. Moreover, hyperglycemia leads to increased formation of advanced glycation end-products, which can result in pathologic collagen cross-linking and alter the viscoelastic properties of blood vessels [32,33]. Hyperglycemia and hyperinsulinemia both activate the renin–angiotensin system and angiotensin type 2 receptor expression in blood vessels, which results in vessel wall hypertrophy and fibrosis [33]. Galectin-3 has been found to bind directly to insulin receptors and inhibit downstream cellular pathways, with resultant insulin resistance in multiple organs and related complications [35]. As mentioned previously, impaired insulin sensitivity is one of the key factors contributing to AS.

SBP was associated with AS in the present study comprising patients receiving PD. Our study results also demonstrated that underlying hypertensive disorders were associated with galectin-3 levels, but HTN was not an independent correlate after adjustment of some clinical factors. HTN is a known risk factor for AS; in contrast, AS can also contribute to arterial hypertension. The pathophysiology of AS includes HTN-included extracellular matrix deposition, activation of focal adhesion complexes, activation of the renin–angiotensin system, actin polymerization, vascular calcification and inflammation, and VSMC proliferation and differentiation into osteoblastic-like cells [36,37]. The causative role of galectin-3 in inducing high blood pressure is not known, but, as mentioned above, HTN may be secondary to vascular remodeling due to the effect of galectin-3.

Blood vessels stiffen with increasing age. The mechanisms underlying arterial aging include endothelial dysfunction, atherosclerosis, matrix remodeling with fewer elastin fibers and increased collagen fibers, deposition of advanced glycation end-products and calcium content, and the presence of comorbid conditions such as hypertension and DM [38,39].

Male gender is also reportedly an independent predictor of AS in patients receiving PD. The mechanisms underlying this association remain unknown; however, female sex hormones are cardioprotective and have been shown to modulate AS [40]. Estrogen may slow progression of atherosclerosis by increasing high-density lipoprotein, decreasing LDL, decreasing lipoprotein(a), enhancing the release of nitric oxide, and inhibiting monocyte migration and adhesion to endothelium [41]. There is greater increase in AS among perimenopausal and postmenopausal women compared with premenopausal females [42]. We did not investigate the menstrual status of the study participants, but the vast majority of female patients might be in postmenopausal state based on their age profiles. Testosterone deficiency may be associated with arterial stiffening in both men and women; the proposed mechanisms include proinflammatory cytokine upregulation, oxidative stress, and subsequent induction of endothelial dysfunction [43]. However, serum testosterone levels were not evaluated in our study.

On the other hand, markers of CKD-mineral and bone disorder (CKD-MBD) including calcium, phosphorus, and iPTH did not differ significantly between the AS and non-AS groups. Although vascular calcification is related to disturbed calcium and phosphorus homeostasis, serum levels of calcium and phosphorus might not be fully representative of the extent of medial calcification. Studies focusing on the association between CKD-MBD markers and AS have shown conflicting results. In a cross-sectional study, serum calcium and phosphorus concentrations were not an independent predictor of AS among CKD patients [44]. Another small study showed that, in patients undergoing peritoneal dialysis, aortic PWV were not correlated with serum calcium, phosphorus, or iPTH levels [45]. In a study recruiting participants with normal kidney function, high–normal or abnormally high serum phosphorus levels were found to be positively associated with brachial-ankle PWV [46].

Solute clearance, which was measured with Kt/V and creatinine clearance, was similar between the two study groups. One explanation is that the determination of dialysis efficiency is based on small solutes rather than middle-molecular-weight molecules. Certain middle-molecular-weight uremic solutes, which include β2-microglobulin, inflammatory biomarkers, leptin, and advanced glycosylation end products, are either poorly dialyzed or not dialyzed. Hemofiltration by utilizing convective solute transport or kidney transplantation might reverse or retard progression of AS [24,47].

As noted in the results of the present research, galectin-3 was not associated with certain variables such as age, biomarkers of CKD-MBD, or total solute clearance, although these may be the fundamental factors in the pathophysiology of AS. The findings might indicate that galectin-3 leads to AS through undetermined mechanisms other than high blood pressure, hyperinsulinemia, and hyperglycemia. Furthermore, the C-statistic only increased slightly after the addition of galectin-3 to certain AS risk factors. Further animal and clinical studies are warranted to further investigate the underlying mechanisms as well as to determine galectin-3 as an accurate predictor for AS.

There were a number of limitations in the current investigation. First, despite the fact that there were patients from four hospitals, the sample size was somewhat relatively small. Second, because this was a cross-sectional investigation, we were unable to investigate the causal link between serum galectin-3 levels and AS. Third, patients receiving PD do not all concur on what constitutes AS. As mentioned above, we currently define AS using a cfPWV cutoff of >10 m/s. Moreover, determination of the cutoff value of 10 m/s is based on the applanation tonometry measurement rather than the XCEL cuff-based device used in the present study. However, the difference between the PWV values measured with these two devices is minimal after adjustment; the XCEL device has thus been validated for clinical measurement of AS [48,49]. Fourth, the AUC for galectin-3 was below 0.7, making it have a limited capability to discriminate patients with AS from those without AS. Fifth, not all the patients recruited in the study had available data on biomarkers of inflammation, such as white blood cell counts and CRP concentrations. Only 107 of the overall study participants had data on CRP; further analysis of its association with AS and galectin-3 is shown in Supplementary Table S2. Sixth, the study did not include an internal control group comprising patients with normal cfPWV.

There is currently no concrete data showing that higher serum galectin-3 levels are connected to or contribute to reduced survival in this patient population, despite the fact that AS is recognized to be a predictor of poor outcomes among patients receiving PD. The usefulness of galectin-3 levels as a predictor of clinical outcomes will require more research with prospective, longitudinal designs. Furthermore, by using animal models to explore the state of galectin-3 mRNA and protein expression at the tissue or vessel wall level, we may have better understanding of the underlying mechanisms of galectin-3 in atherosclerotic processes, as well as in CV morbidities.

## 5. Conclusions

Serum galectin-3 levels were positively and independently connected with cfPWV, a biomarker of AS, in the current investigation, which included patients with ESKD receiving PD.

## Figures and Tables

**Table 1:** Baseline characteristics of patients.

Characteristic	All Participants (*n* = 196)	Group without Aortic Stiffness (*n* = 148)	Aortic Stiffness Group (*n* = 48)	*p* Value
Age (years)	58.50 ± 14.06	56.94 ± 14.67	63.31 ± 10.75	0.006 *
Female, *n* (%)	109 (55.6)	93 (62.8)	16 (33.3)	<0.001 *
Diabetes, *n* (%)	77 (39.3)	46 (31.1)	31 (64.6)	<0.001 *
Hypertension, *n* (%)	140 (71.4)	100 (67.6)	40 (83.3)	0.036 *
Peritoneal dialysis vintage (months)	49.00 (21.97–83.30)	43.50 (19.71–81.66)	53.34 (29.00–100.95)	0.084
Body mass index (kg/m^2^)	25.02 ± 4.16	24.78 ± 4.26	25.76 ± 3.76	0.160
Waist circumference (cm)	92.44 ± 10.72	91.10 ± 10.93	96.56 ± 8.95	0.002 *
Carotid–femoral PWV (m/s)	9.15 ± 1.60	8.45 ± 1.02	11.30 ± 1.06	<0.001 *
Systolic blood pressure (mm Hg)	149.15 ± 22.11	147.28 ± 21.77	154.90 ± 22.38	0.038 *
Diastolic blood pressure (mm Hg)	85.01 ± 15.01	84.68 ± 15.47	86.00 ± 13.57	0.598
Total cholesterol (mg/dL)	170.24 ± 43.03	173.11 ± 45.44	161.40 ± 33.44	0.101
Fasting glucose (mg/dL)	103.00 (92.00–128.00)	100.00 (91.00–116.00)	121.00 (96.50–166.25)	<0.001 *
Albumin (g/dL)	3.56 ± 0.36	3.57 ± 0.35	3.53 ± 0.39	0.447
Blood urea nitrogen (mg/dL)	62.92 ± 20.70	62.47 ± 20.06	64.33 ± 22.71	0.588
Creatinine (mg/dL)	10.76 ± 3.04	10.74 ± 3.18	10.84 ± 2.62	0.829
Total calcium (mg/dL)	9.61 ± 0.72	9.55 ± 0.72	9.79 ± 0.71	0.053
Phosphorus (mg/dL)	5.26 ± 1.31	5.33 ± 1.36	5.05 ± 1.15	0.201
Calcium–phosphorus product (mg^2^/dL^2^)	50.53 ± 12.96	50.89 ± 13.38	49.39 ± 11.64	0.486
Intact parathyroid hormone (pg/mL)	242.20 (102.51–454.50)	242.20 (106.50–446.88)	234.46 (84.83–511.88)	0.763
Galectin-3 (ng/mL)	82.61 ± 15.97	80.56 ± 15.54	88.92 ± 15.78	0.001 *
Weekly Kt/V	2.07 ± 0.45	2.11 ± 0.48	1.97 ± 0.35	0.061
Peritoneal Kt/V	46.93 ± 13.47	1.86 ± 0.48	1.82 ± 0.36	0.575
Total clearance of creatinine (L/week)	58.78 ± 17.83	58.72 ± 18.32	58.96 ± 16.42	0.936
Peritoneal clearance of creatinine (L/week)	46.93 ± 13.47	46.05 ± 13.73	49.62 ± 12.41	0.111
CAPD, *n* (%)	72 (36.7)	56 (37.8)	16 (33.3)	0.574
ARB use, *n* (%)	124 (63.3)	95 (64.2)	29 (60.4)	0.638
β-blocker use, *n* (%)	93 (47.4)	70 (47.3)	23 (47.9)	0.940
CCB use, *n* (%)	113 (57.7)	86 (58.1)	27 (56.3)	0.821

Abbreviations: CAPD, continuous ambulatory peritoneal dialysis; Weekly Kt/V, weekly fractional clearance index for urea; ARB, angiotensin-receptor blocker; CCB, calcium-channel blocker. * *p* < 0.05 was considered statistically significant.

**Table 2:** Multivariate risk factors correlated to arterial stiffness among 196 patients undergoing peritoneal dialysis.

Variables	Odds Ratio	95% Confidence Interval	*p* Value
Galectin-3, 1 ng/mL	1.029	1.002–1.057	0.034 *
Age, 1 year	1.044	1.011–1.078	0.008 *
Male	3.479	1.548–7.818	0.003 *
Waist circumference, 1 cm	1.019	0.978–1.062	0.368
Diabetes, present	2.142	0.860–5.335	0.102
Hypertension, present	2.215	0.722–6.793	0.164
Systolic blood pressure, 1 mmHg	1.004	0.983–1.025	0.700
Fasting glucose, 1 mg/dL	1.005	0.995–1.015	0.348

Analysis was carried out using the multivariate logistic regression analysis (adopted factors: sex, diabetes, hypertension, waist circumference, age, systolic blood pressure, fasting glucose, and galectin-3). * *p* < 0.05 was considered statistically significant.

**Table 3:** Correlation between carotid–femoral pulse wave velocity levels and clinical variables.

Variables	Carotid–Femoral Pulse Wave Velocity (m/s)				
	Simple Regression		Multivariate Regression		
	*r*	*p* Value	Beta	Adjusted R^2^ Change	*p* Value
Female	−0.330	<0.001 *	−0.309	0.089	<0.001 *
Diabetes	0.387	<0.001 *	0.257	0.161	<0.001 *
Hypertension	0.123	0.086	–	–	–
Age (years)	0.338	<0.001 *	0.306	0.082	<0.001*
Log-PD vintage (months)	0.056	0.433	–	–	–
Body mass index (kg/m^2^)	0.216	0.002 *	–	–	–
Waist circumference (cm)	0.332	<0.001 *	–	–	–
Systolic blood pressure (mm Hg)	0.250	<0.001 *	0.184	0.028	0.002 *
Diastolic blood pressure (mm Hg)	0.052	0.468	–	–	–
Total cholesterol (mg/dl)	−0.126	0.078	–	–	–
Log-Glucose (mg/dL)	0.372	<0.001*	–	–	–
Albumin (g/dL)	−0.077	0.284	–	–	–
Blood urea nitrogen (mg/dL)	0.011	0.880	–	–	–
Creatinine (mg/dL)	−0.014	0.841	–	–	–
Total calcium (mg/dL)	0.110	0.126	–	–	–
Phosphorus (mg/dL)	−0.126	0.078	–	–	–
Calcium–phosphorus product (mg^2^/dL^2^)	−0.090	0.207	–	–	–
Log-iPTH (pg/mL)	−0.050	0.489	–	–	–
Galectin-3 (ng/mL)	0.274	<0.001 *	0.184	0.031	0.001 *
Log-Weekly Kt/V	−0.101	0.159	–	–	–
Peritoneal Kt/V	−0.050	0.484	–	–	–
Total clearance of creatinine (L/week)	0.022	0.763	–	–	–
Peritoneal clearance of creatinine (L/week)	0.133	0.067	–	–	–

Abbreviations: PD, peritoneal dialysis; iPTH, intact parathyroid hormone; Weekly Kt/V, weekly fractional clearance index for urea. * *p* < 0.05 was considered statistically significant.

**Table 4:** Correlation between serum galectin-3 levels and clinical variables.

Variables	Serum Galectin-3 (ng/mL)				
	Simple Regression		Multivariate Regression		
	*r*	*p* Value	Beta	Adjusted R^2^ Change	*p* Value
Female	−0.067	0.352	–	–	–
Diabetes	0.146	0.041 *	–	–	–
Hypertension	0.160	0.025 *	–	–	–
Age (years)	0.032	0.657	–	–	–
Log-PD vintage (months)	0.111	0.121	–	–	–
Body mass index (kg/m^2^)	0.068	0.342	–	–	–
Waist circumference (cm)	0.019	0.788	–	–	–
Carotid–femoral PWV (m/s)	0.212	0.003 *	2.741	0.070	<0.001 *
Systolic blood pressure (mm Hg)	0.070	0.327	–	–	–
Diastolic blood pressure (mm Hg)	−0.033	0.649	–	–	–
Total cholesterol (mg/dl)	0.025	0.727	–	–	–
Log-Glucose (mg/dL)	0.161	0.024 *	–	–	–
Albumin (g/dL)	0.070	0.332	–	–	–
Blood urea nitrogen (mg/dL)	0.003	0.969	–	–	–
Creatinine (mg/dL)	0.038	0.596	–	–	–
Total calcium (mg/dL)	0.060	0.407	–	–	–
Phosphorus (mg/dL)	−0.096	0.181	–	–	–
Calcium–phosphorus product (mg^2^/dL^2^)	−0.060	0.404	–	–	–
Log-iPTH (pg/mL)	−0.028	0.701	–	–	–
Log-Weekly Kt/V	0.077	0.281	–	–	–
Peritoneal Kt/V	0.146	0.041 *	–	–	–
Total clearance of creatinine (L/week)	−0.041	0.573	–	–	–
Peritoneal clearance of creatinine (L/week)	0.037	0.611	–	–	–

Data on PD vintage, glucose, iPTH levels, and weekly Kt/V showed skewed distribution, and therefore were log-transformed before analysis. Analysis was performed using simple regression analysis or multivariable stepwise linear regression analysis (adopted factors: female, diabetes, age, body mass index, waist circumference, systolic blood pressure, log-glucose, and galectin-3). Abbreviations: PD, peritoneal dialysis; PWV, pulse wave velocity; iPTH, intact parathyroid hormone; Kt/V, fractional clearance index for urea. * *p* < 0.05 was considered statistically significant.

## Data Availability

The data presented in this study are available on request from the corresponding author.

## Supplementary Materials

The following supporting information can be downloaded at: https://www.mdpi.com/article/10.3390/jcm12103519/s1. Table S1: The C-statistic discrimination tests of clinical variables with and without galectin-3 on the correlation with AS. Table S2: Spearman’s correlation analysis of the association of C-reactive protein with galectin-3 and aortic stiffness.
