# Supplementary material for: Positive Association of Serum Galectin-3 with the Development of Aortic Stiffness of Patients on Peritoneal Dialysis
Source: J Clin Med. 2023 May 17;12(10):3519. doi: 10.3390/jcm12103519 (PMC10218976; doi:10.3390/jcm12103519)
Supplement: Supplementary file 1 [file jcm-12-03519-s001.zip › jcm-2332290-supplementary.pdf]

Supplementary Table S1. The C-statistic discrimination tests of clinical variables with and without galectin-3 on the correlation with AS.

Models	C-statistic	95% CI	P value
Age, gender, SBP, and DM	0.778	0.713 to 0.834	0.318
Age, gender, SBP, and DM + galectin-3	0.795	0.731 to 0.849	

Abbreviations: CI, confidence interval; SBP, systolic blood pressure; DM, diabetes mellitus.

Supplementary Table S2. Spearman's correlation analysis of the association of C-reactive protein with galectin-3 and aortic stiffness.

		CRP	GAL3	PWV
CRP	r<br>P<br>n		0.282<br>0.0032*<br>107	0.175<br>0.0721<br>107
GAL3	r<br>P<br>n	0.282<br>0.0032*<br>107		0.212<br>0.0029*<br>196
PWV	r<br>P<br>n	0.175<br>0.0721<br>107	0.212<br>0.0029*<br>196	

Only 107 of the overall study participants had available data on CRP concentrations. Although CRP appeared to be positively associated with galectin-3 levels, it did not show a significant correlation with PWV, which is a marker of aortic stiffness. Abbreviations: CRP, C-reactive protein; GAL3, galectin-3; PWV, pulse wave velocity. *$P < 0.05$ was considered statistically significant.
